# Acquired Dyslexia in a Transparent Orthography: An Analysis of Acquired Disorders of Reading in the Slovak Language

**DOI:** 10.3233/BEN-2012-119005

**Published:** 2012-03-19

**Authors:** Marianna Hricová, Brendan Stuart Weekes

**Affiliations:** ^1^Ludwig Maximilian University of MunichMunichGerman; ^2^University of Hong KongHong KongChina

**Keywords:** Acquired dyslexia, phonological dyslexia, surface dyslexia, deep dyslexia, dyslexia in the Slovak language, orthographic transparency, Slovak orthography

## Abstract

The first reports of phonological, surface and deep dyslexia come from orthographies containing quasi-regular mappings between orthography and phonology including English and French. Slovakian is a language with a relatively transparent orthography and hence a mostly regular script. The aim of this study was to investigate impaired oral reading in Slovakian. A novel diagnostic procedure was devised to determine whether disorders of Slovakian reading resemble characteristics in other languages. Slovakian speaking aphasics showed symptoms similar to phonological dyslexia and deep dyslexia in English and French, but there was no evidence of surface dyslexia. The findings are discussed in terms of the orthographic depth hypothesis.

## 1. Introduction

Alphabetic writing systems differ with regard to how consistently letters map onto sound. In relatively consistent orthographies such as German, Greek, Italian, Spanish or Turkish, letters or letter groups usually map to the same sound in different words. However, in inconsistent orthographies such as English and French, the relation between letters and sound is variable i.e., some letters or letter clusters can be pronounced in more than one way and some sounds spelled in different ways. Languages with inconsistent grapheme to phoneme correspondences are referred to as opaque or deep orthographies and languages with regular and consistent grapheme to phoneme correspondences as shallow or transparent orthographies.

The transparency of the correspondences between phonemes and graphemes may have an impact on word recognition. The orthographic depth hypothesis [20, 24] assumes that oral reading varies according to orthographic transparency in terms of dual pathway accounts of reading [4]. Specifically orthographic transparency will determine the relative contribution of lexical-semantic and sublexical mechanisms used to read aloud. The strongest version of the orthographic depth hypothesis assumes that a lexical-semantic reading mechanism is redundant in transparent orthographies. This is because the sublexical reading mechanism will always give a correct pronunciation for all letter strings. One prediction of this hypothesis is that semantic reading errors e.g. symphony → *orchestra* that reflect a lexical-semantic reading mechanism, as well as regularization errors where irregularly spelled words such as *yacht* are misread, will not be observed in transparent orthographies. However some patients with acquired dyslexia in transparent orthographies including Greek, Italian, Spanish and Turkish do show these errors [4,10–12,17,18,22,23,29,33,37]. Slovakian is also a transparent orthography. However little is known about disorders of reading in Slovakian. The aim of this study is to test the orthographic depth hypothesis

ISSN 0953-4180/12/$27.50 © 2012 – IOS Press and the authors. All rights reserved

Table 1
Graphemes and phonemes in Slovak language

| Grapheme | Phoneme (IPA)* | Phoneme Slovak transcription | Grapheme | Phoneme (IPA)* | Phoneme Slovak transcription |
|---|---|---|---|---|---|
| a | a | a | m | m | m |
| á | aː | á | n | n | n |
| ä | æ, ɛ | ä, e | ň | ɲ, nʲ | ň |
| b | b | b | o | ɔ | o |
| c | t͡s | c | ó | ɔː | ó |
| č | t͡ʃ | č | ô | u̯o | ŭo |
| d | d | d | p | p | p |
| ď | ɟ, dʲ | ď | q | kv | kv |
| dz | d͡z | ʒ | r | r, r̩ | r |
| dž | d͡ʒ | ǯ | ŕ | r̩ː | ŕ̥ |
| e | e | e | s | s | s |
| é | eː | é | š | ʃ | š |
| f | f | f | t | t | t |
| g | g | g | ť | c, tʲ | ť |
| h | ɦ | h | u | u | u |
| ch | x | x | ú | uː | ú |
| i | ɪ | i | v | v | v |
| í | iː | í | w | v | v |
| j | j | j | x | ks | ks |
| k | k | k | y | ɪ | i |
| l | l, l̩ | l | ý | iː | í |
| ĺ | l̩ː | ĺ̥ | z | z | z |
| ľ | ʎ, lʲ | ľ | ž | ʒ | ž |

* International phonetic alphabet.

by surveying the types of acquired dyslexia found in Slovakian speakers with aphasia.

## 2. Slovak script

The number of grapheme to phoneme correspondences in Slovakian orthography is not as well defined as in English [25] and Italian [24]. However, if the degree of language regularity is defined as the percentage of words for which pronunciation does not correspond to the pronunciation rules [38] then Slovakian can be considered a transparent orthography. Table 1 provides a summary of phoneme-grapheme correspondences in Slovak. There are 13 vowels, 32 consonants (four consonants are in certain cases considered as vowels) and four diphthongs [14]. Slovak orthography is based on several principles: the phonemic principle; the morphological principle; and the grammatical principle [6]. The phonemic principle of Slovak spelling allows proficient spelling based on sound alone. This rule also applies to oral reading whereby mappings from spelling to sound follow one to one grapheme-to-phoneme correspondence. Although this principle applies to most graphemes, there are some graphemes that are represented by more than one phoneme. For example, <b> could be pronounced [b] on the initial word position or [p] final; <d> could be pronounced [d] initial or [t] final; <d'> as [d] initial or [t] on the final word position. Similarly <z> can be pronounced as [z] initial or [s] final and <ž> as [ʒ] initial or [ʃ] final. Exceptions are qualified by an assimilation rule. The grapheme <n> can be represented by two phonemes varying in pronunciation: alveolar articulation is used if <n> is at the start of a word or between two vowels, and velar articulation if <n> is followed by a consonant.

For other letters, application of grapheme-phoneme rules is problematic e.g. the consonants <t>, <d>, <n>, <l>, which are pronounced [t], [d], [n], [l] are pronounced differently if followed by <i>, <í>, <e>, <ia>, <ie> or <iu>. This may require some lexical knowledge. Similarly, foreign and loan words are present in Slovakian and these often have irregular pronunciations. Although the rule "read <te>, <de>, <ne>, <le> softly" can be applied to words of foreign origin or to relatively unknown proper nouns, this rule is often not applied. Instead hard pronunciation of the syllables in those words is common. Such words could be labeled irregular in Slovak, as they come from languages with less transparent orthography typically English and French. Reading of foreign loan words and proper nouns normally relies on a lexical reading mechanism although there is no direct evidence to support the existence of such a reading mechanism in Slovakian. Given that extant models of reading in transparent scripts such as Spanish do include a lexical-semantic pathway (e.g. Iribarren et al. [22]) it is in principle possible that damage to a lexical reading mechanism for Slovakian might result in over-reliance on grapheme-phoneme rules in reading thus producing regularization errors in Slovakian [2].

## 3. Acquired dyslexia in transparent orthographies and in Slovakian

The different forms of acquired dyslexia reported in English and French are called central dyslexias [35].

These are deep dyslexia, phonological dyslexia and surface dyslexia. The characteristic feature of deep dyslexia is the production of semantic paralexias – semantic errors in reading aloud indicate the use of a lexical-semantic reading mechanism [8]. Deep dyslexics also produce visual errors. In most cases nonword reading is also impaired, indicating an additional impairment to a sub-lexical reading mechanism. Further, deep dyslexics often misread abstract words or function words. Reading of verbs and adjectives is also poor, but better than function words. Morphological errors are also produced [32]. Accounts of disturbed reading in deep dyslexia include the right hemisphere hypothesis [7] and poor reading resulting from damage to an integrated nonlexical reading mechanism i.e. the deep-phonological dyslexia continuum [9].

The orthographic depth hypothesis assumes that the predictability of the mappings between orthography and phonology will influence reading performance and the division of labour for oral reading in transparent orthographies relies on nonlexical reading mechanisms. It therefore follows that reading disorders in Slovakian will primarily reflect damage to the nonlexical reading mechanism resulting in poor word and nonword reading. Therefore phonological dyslexia which describes poor nonword reading with intact word reading should not be observed. An additional prediction is that semantic reading errors in deep dyslexia, which characterizes reading via the lexical-semantic pathway, should not be observed. However, contrary to this prediction deep dyslexia has been reported in languages with a transparent orthography [10,12,33]. For example, Ferreres and Miravalles [18] reported a Spanish-speaking patient whose production of semantic errors was remarkably similar to descriptions of deep dyslexia in English by Marshall and Newcombe [28]. Cases of deep dyslexia have also been reported in other languages with relatively transparent orthography such as Italian [3] Greek [17] and Welsh [4] suggesting that a transparent writing system does not necessarily limit the production of semantic paralexias. In Slovak, Marková [27] described a patient who produced visual paralexias and disturbed reading of nonwords that was similar to the characteristics of deep dyslexia. As in languages with more opaque orthographies such as French [13] and English [31], therapy to improve sublexical reading also resulted in improved lexical reading. These results suggest that deep dyslexia in Slovakian reflects use of lexical-semantic reading mechanisms lead Marková to question whether intact functioning of a sub-lexical mechanism would be sufficient to read in Slovakian.

Phonological dyslexia was first identified in a French-speaker [5] and subsequently reported in English [21]. This reading disorder refers to impaired nonword reading with word reading preserved. A characteristic symptom is visual reading errors. Phonological dyslexia is also observed in languages with transparent orthography such as Spanish [11,22], Italian [37] and Japanese kana [34]. Phonological dyslexia has been described in a Slovak-speaking patient [15] who had selective problems with nonword reading and produced phonological paralexias. This suggested a selective dysfunction to the sublexical reading mechanism but more critically, a dissociation showing that the lexical reading mechanism appeared to be reading words normally.

The reverse dissociation called surface dyslexia was also first reported in French and English [28]. This refers to a impairment to the lexical reading mechanism. Patients read nonwords and words with regular spellings well, but reading of irregular words results in regularisation errors. The identification of surface dyslexia in languages with transparent orthographies is controversial. If all words can be read correctly using grapheme to phoneme correspondence rules then surface dyslexia should not exist. However regularisation errors occur when reading foreign words or loanwords in these languages as these must be read via lexical reading mechanisms [1]. Some patients with acquired dyslexia in Italian and German also produce incorrect stress patterns during oral reading and these errors are considered to be regularizations because they reflect the operation of a lexical reading mechanism [23,37]. Although there are no reports of surface dyslexia in Slovakian, the suggestion by Marková [27] that reading in Slovakian can proceed using a lexical reading pathway raises the possibility that surface dyslexia for oral reading of foreign words may be observed

## 4. Aims

The motivation for the present study was to examine the patterns of acquired dyslexia observed in Slovak speakers with the following aims: (1) to compile diagnostic material for assessment of the performance of Slovak-speaking stroke patients in oral reading; and (2) to obtain oral reading data for Slovak-speaking stroke patients and (3) to analyse these error data for the presence of symptoms found in cases of acquired dyslexia in other languages reported above.

Table 2
Dempgraphic information

| Patient | Age | Sex | Years of education | Mother tongue | Bilingualism | Laterality | Speech diagnosis | Stage of illness |
|---|---|---|---|---|---|---|---|---|
| 1. J.S. | 57 | M | 18 | S | – | R | NC | A |
| 2. ŠU. | 80 | F | 13 | S | – | R | AA | A |
| 3. J.S. | 76 | M | 13 | S | – | R | NC | C |
| 4. H.J. | 73 | F | 13 | S | – | R | AA | C |
| 5. K.Ž | 71 | M | 13 | S | – | R | AA | S |
| 6. D.CH. | 63 | M | 13 | S | – | R | TMA | A |
| 7. K.J. | 45 | M | 13 | S | – | R | AA | A |
| 8. E.K. | 82 | F | 9 | A | S-H | R | AA | A |
| 9. K.Č | 78 | M | 18 | S | – | R | AA | C |
| 10. U.Š | 84 | F | 13 | S | – | R | AA | C |
| 11. H.M. | 63 | M | 13 | S | – | R | AA | S |
| 12. K.F. | 79 | M | 18 | S | – | R | AA | S |
| 13. R.B. | 75 | M | 18 | S | – | R | BA | S |
| 14. N.O. | 74 | F | 13 | A | S-H | R | AA | S |
| 15. C.A. | 78 | M | 13 | A | S-unk. | R | BA | A |
| 16. S.J. | 52 | M | 18 | S | – | R | BA | A |
| 17. B.K. | 43 | F | 13 | S | – | R | BA | A |
| 18. O.L. | 86 | F | 13 | S | – | A | BA | A |
| 19. O.L' | 47 | M | 13 | S | – | R | BA | C |
| 20. M.H. | 63 | M | 18 | S | – | R | BA | C |
| 21.J.U. | 74 | M | 9 | S | – | R | NC | A |
| 22. K.M. | 75 | F | 13 | S | – | A | CA | A |
| 23. M.B. | 75 | F | 9 | S | S-B | R | CA | A |
| 24. R.V. | 73 | F | 9 | A | S-H | R | CA | C |
| 25. J.K. | 51 | M | 18 | S | – | R | CA | S |
| 26. J.S. | 73 | M | 13 | S | – | R | CA | S |
| 27. J.S. | 73 | M | 13 | S | – | R | TMA | C |
| 28. Z.Š | 52 | F | 18 | S | – | R | TMA | C |
| 29. J.H. | 56 | M | 13 | S | – | R | WA | A |
| 30. I.B. | 41 | F | 13 | S | – | R | WA | S |

Mother tongue: S-Slovak language, A-another; Bilingualism: S-H Slovakian-Hungarian, S-B Slovakian-Bulgarian, S-unk. Slovakian-second language unknown; Laterality: R-right-handed, L-left-handed, A-ambidextral; Speech diagnosis: BA-Broca Aphasia, WA-Wernicke Aphasia, CA-Conduction Aphasia, AA-Anomic Aphasia, TMA-Transcortical Motor Aphasia, TSA-Transcortical Sensory Aphasia, NC-Non-classifiable Aphasia; Stage of illness: A-Acute (< 4 weeks), S-Subacute (> 4 weeks < 6 months), C-Chronic (> 6 months).

## 5. Method

### 5.1. Participants

30 participants with cerebro-vascular disease. Of these, 18 (60%) were male aged 45–79 years and 12 (40%) were female aged 41–86 years. Four participants were bilingual. 28 patients (93.34%) were classified as right-handed and two (6.67%) as ambidextrous. The educational achievement of participants was variable ranging from primary education (9 years), $n = 4$ (13.34%) to secondary education (13 years), $n = 18$ (60%) and university education (18 years), $n = 8$ (26.67%). Table 2 provides demographic data. Speech assessment was based on the Boston aphasia classification.

### 5.2. Tasks

Assessment of reading disorder was designed to elicit the symptoms reported in other transparent orthographies. Tasks were designed so that each measured the presence of one symptom, i.e. an effect of word-length (high frequency nouns matched to word-length); a word-class-effect (nouns, adjectives, verbs, functors matched to frequency of occurrence and no longer than three-syllable); a concreteness effect (high frequency concrete and abstract nouns no longer than three-syllable); a lexicality effect measured with two forms of pseudoword (Type A – pseudowords – in legible Slovak letter sequences, and Type B – pseudowords, created by changing one letter from a real Slovak word. Reading of pseudohomophones – letter strings that differ in their orthographic but not their phonological form to a real word cannot be tested in Slovakian); a regularity effect whereby foreign and loan words to which Slovak reading rules do not apply were classified as irregular; and tests of semantic processing. All tasks were presented to 30 control participants including 16 males aged 45 to 84 and 14 females age 54 to 82 with primary education $n = 3$ (10%), secondary education n=16 (53.33%) and university education $n = 11$ (36.66%). Performance was at ceiling for most tasks with only irregular word reading showing difficulty with scores ranging from 70–90% correct.

## 6. Results

The reading performance of each participant was examined for the presence of typical symptoms of acquired dyslexia. These results are summarized in Table 3.

Poor performance on nonword reading tasks compared to word reading tasks is assumed to reflect specific impairment to sub-lexical reading mechanisms and if nonword reading is worse than word reading in addition to problems with reading function words, effects of part of speech, concreteness and word frequency then a classification of phonological dyslexia would be considered. Visual errors would be further indicators of phonological dyslexia. Note that this pattern of reading is also observed in deep dyslexia but with more severe impairment [9] including the production of semantic paralexias. Semantic errors will therefore be an indication of deep dyslexia. Poor performance on irregular (foreign and loan) word reading compared to nonword reading is assumed to reflect malfunctioning of a lexical reading mechanism and so if foreign and loan word reading is worse than nonword reading, then a classification of surface dyslexia would be considered. Regularisation errors when reading these items would also be further indicators of surface dyslexia.

*Nonword reading*: Impaired nonword reading was observed in several patients and controls scored 100% correct. For 14 patients (46.66% of total sample) overall performance was below 30%. There was a discrepancy between word reading and nonword reading for 6 cases (case numbers 4, 6, 10, 16, 21, 22 shown in Table 3) whereby word reading was at least 84% correct and nonword reading performance was below 15% correct. An additional four cases (numbers 3, 8, 13, 24 shown in Table 3) showed a pattern whereby word reading was at least twice as proficient as reading performance with nonwords. Therefore, at least 20 patients show evidence of phonological dyslexia in Slovakian.

*Part of speech:* Only one case (number 4) showed selectively poor reading of function words (20% correct). This case also had impaired nonword reading as noted above, and is therefore compatible with the classification of phonological dyslexia.

*Concreteness/Abstractness*: There was no evidence of concreteness/imageability effects on reading for any participant.

*Frequency of occurrence*: There was no evidence of an effect of frequency on reading for any participant.

*Semantic and visual paralexias*: Many patients produced at least one semantic error. However no patient produced more than five semantic errors, which is a relatively small number. Only one participant (number 4 in Table 3) produced semantic as well as visual errors and read nonwords, function words and low frequency words poorly (with no difference in reading concrete and abstract words). Thus one participant showed evidence of deep dyslexia in Slovakian.

*Irregular words:* One participant (number 20 in Table 3) showed impaired reading of irregular words (50% correct) that was below the level achieved by controls. However this case also read nonwords poorly. Therefore, no participant could be classified as surface dyslexic on the basis of dissociation between irregular word and nonword reading.

## 7. Discussion

The aim of this study was to assess whether acquired phonological, deep and surface dyslexia would be observed in Slovakian a language with a transparent orthography. In less transparent languages such as English and French selective damage to both lexical-semantic and sub-lexical reading mechanisms can be assumed. However in languages with transparent orthography use of a lexical-semantic reading mechanism is controversial. The present results show that word reading can be preserved in Slovakian when nonword reading (via a sublexical mechanism) is impaired. This is a novel finding but is compatible with the results from other transparent scripts such as Spanish, Italian and German. The results are also compatible with the suggestion by Marková [27] that reading in Slovakian can proceed using at least two pathways: a sublexical mechanism which is damaged in phonological and deep dyslexia and a lexical reading pathway which is preserved.

Table 3

Results of individual participans (accuracy in %)

| Patient | One-syllable words | Two-syllable words | Three-syllable words | Four-syllable words | Five/six-syllable words | Nouns | Adjec-tives | Verbs | Func-tors | Seman-tic task | Catego-risation | Concrete nouns | Abstract nouns | High-frequent | Low-frequent | Pseudo-words A-type | Pseudo-words B-type | Irregular words | Seman-errors (count) | Orthographic errors (count) |
|---|---|---|---|---|---|---|---|---|---|---|---|---|---|---|---|---|---|---|---|---|
| 1. J.S. | 75 | 75 | 100 | 100 | 75 | 100 | 100 | 100 | 80 | 100 | 100 | 80 | 90 | 90 | 80 | 90 | 100 | 100 | – | – |
| 2. ŠU. | 100 | 100 | 100 | 100 | 100 | 100 | 100 | 100 | 100 | 90 | 70 | 100 | 90 | 100 | 100 | 60 | 100 | 90 | 1 | – |
| 3. J.S. | 75 | 50 | 100 | 75 | 100 | 80 | 80 | 60 | 80 | 70 | 100 | 80 | 90 | 90 | 90 | 30 | 20 | 90 | – | 3 |
| 4. H.J. | 100 | 50 | 100 | 75 | 100 | 80 | 60 | 80 | 20 | 80 | 100 | 70 | 60 | 100 | 70 | 0 | 10 | 90 | 2 | – |
| 5. K.Ž | 100 | 100 | 100 | 100 | 100 | 100 | 100 | 100 | 100 | 80 | 80 | 100 | 90 | 100 | 80 | 70 | 70 | 90 | 2 | – |
| 6. D.CH. | 75 | 100 | 75 | 100 | 100 | 80 | 100 | 80 | 60 | 80 | 50 | 70 | 80 | 100 | 70 | 20 | 0 | 100 | 2 | – |
| 7. K.J. | 75 | 100 | 100 | 100 | 100 | 100 | 100 | 100 | 100 | 100 | 100 | 100 | 100 | 100 | 100 | 50 | 60 | 100 | – | – |
| 8. E.K. | 75 | 100 | 75 | 100 | 100 | 100 | 100 | 100 | 100 | 40 | 30 | 100 | 100 | 90 | 80 | 60 | 20 | 100 | 3 | 3 |
| 9. K.Č | 100 | 100 | 100 | 100 | 100 | 100 | 80 | 100 | 100 | 100 | 90 | 100 | 100 | 100 | 100 | 80 | 80 | 100 | – | – |
| 10. U.Š | 100 | 100 | 100 | 75 | 75 | 100 | 100 | 80 | 100 | 70 | 80 | 100 | 80 | 100 | 90 | 10 | 20 | 90 | 2 | 1 |
| 11. H.M. | 100 | 100 | 100 | 75 | 100 | 80 | 100 | 100 | 100 | 90 | 90 | 80 | 50 | 80 | 90 | 40 | 60 | 100 | 1 | – |
| 12. K.F. | 100 | 100 | 100 | 100 | 75 | 100 | 100 | 100 | 100 | 80 | 70 | 100 | 90 | 100 | 90 | 50 | 60 | 100 | 1 | 1 |
| 13. R.B. | 100 | 100 | 100 | 100 | 100 | 80 | 100 | 100 | 100 | 80 | 100 | 100 | 100 | 90 | 90 | 20 | 50 | 100 | 2 | – |
| 14. N.O. | 100 | 100 | 100 | 100 | 100 | 100 | 100 | 100 | 100 | 100 | 100 | 100 | 100 | 90 | 80 | 70 | 80 | 100 | – | – |
| 15. C.A. | 100 | 100 | 75 | 50 | 25 | 60 | 100 | 100 | 60 | 70 | 100 | 50 | 60 | 80 | 60 | 30 | 40 | 100 | 3 | – |
| 16. S.J. | 75 | 100 | 100 | 100 | 100 | 100 | 100 | 60 | 60 | 50 | 50 | 100 | 70 | 90 | 70 | 10 | 10 | 100 | 5 | – |
| 17. B.K. | 100 | 100 | 75 | 75 | 0 | 100 | 100 | 100 | 100 | 70 | 30 | 50 | 70 | 60 | 70 | 0 | 0 | 100 | 3 | – |
| 18. O.L. | 75 | 100 | 100 | 100 | 25 | 100 | 100 | 100 | 100 | 90 | 60 | 100 | 100 | 80 | 100 | 40 | 20 | 100 | 1 | – |
| 19. O.L' | 100 | 75 | 75 | 25 | 0 | 80 | 40 | 0 | 40 | 80 | 80 | 50 | 30 | 70 | 30 | 0 | 0 | 100 | 1 | 1 |
| 20. M.H. | 75 | 75 | 50 | 25 | 50 | 80 | 80 | 60 | 80 | 50 | 60 | 60 | 80 | 70 | 60 | 20 | 40 | 50 | 3 | 1 |
| 21.J.U. | 75 | 100 | 100 | 75 | 100 | 100 | 100 | 100 | 100 | 80 | 0 | 90 | 70 | 90 | 70 | 10 | 20 | 100 | 1 | – |
| 22. K.M. | 75 | 100 | 75 | 75 | 100 | 100 | 80 | 60 | 80 | 80 | 50 | 90 | 80 | 80 | 50 | 0 | 0 | 100 | 2 | – |
| 23. M.B. | 100 | 100 | 100 | 100 | 100 | 100 | 100 | 100 | 100 | 90 | 40 | 100 | 100 | 100 | 100 | 80 | 90 | 80 | – | – |
| 24. R.V. | 100 | 100 | 100 | 75 | 75 | 100 | 80 | 100 | 100 | 80 | 60 | 100 | 90 | 90 | 80 | 40 | 30 | 100 | 2 | – |
| 25. J.K. | 50 | 50 | 50 | 25 | 0 | 40 | 60 | 40 | 100 | 90 | 100 | 60 | 60 | 70 | 70 | 30 | 40 | 70 | – | – |
| 26. J.S. | 100 | 100 | 100 | 75 | 50 | 100 | 80 | 60 | 60 | 80 | 50 | 90 | 70 | 100 | 100 | 50 | 50 | 100 | 2 | – |
| 27. J.S. | 100 | 75 | 100 | 75 | 100 | 80 | 100 | 100 | 100 | 100 | 80 | 90 | 100 | 100 | 90 | 60 | 50 | 100 | – | – |
| 28. Z.Š | 100 | 100 | 100 | 75 | 50 | 80 | 80 | 60 | 100 | 60 | 30 | 100 | 80 | 80 | 100 | 30 | 30 | 80 | 3 | 1 |
| 29. J.H. | 100 | 100 | 100 | 100 | 75 | 100 | 80 | 100 | 80 | 90 | 100 | 100 | 90 | 70 | 90 | 50 | 40 | 90 | 1 | – |
| 30. I.B. | 75 | 100 | 50 | 75 | 25 | 60 | 100 | 80 | 60 | 70 | 40 | 50 | 80 | 50 | 40 | 0 | 40 | 70 | 1 | – |

The results allow two predictions derived from the orthographic depth hypothesis to be rejected. First, lexical-semantic reading mechanism is not redundant in Slovakian and second, semantic reading errors e.g. symphony → *orchestra* should are observed in Slovakian. The present results show that a lexical-semantic reading mechanism is available for reading in Slovakian when the sublexical mechanism is not available and this mechanism generates semantic reading errors. Thus, we contend that reading in Slovakian normally involves a lexical reading mechanism that provides access to meaning allowing reading for meaning and text comprehension and generates semantic errors if the sublexical mechanism is damaged.

An important caveat to our claim is that nonword reading may be a more difficult task than word reading in Slovakian. It is certainly an unfamiliar to most readers in Slovakian. Therefore poor nonword reading may not simply reveal damage to the sublexical reading mechanism but show that brain damage selectively affects oral reading of unfamiliar letter strings. The strongest evidence against this possibility would be reports of the reverse dissociation shown by acquired surface dyslexic readers in English and French i.e. preserved nonword reading with impaired word reading in Slovakian. Surface dyslexia in Slovakian would also provide direct evidence of independent lexical-semantic and sublexical mechanisms for reading to support the claims made by Marková [27]. There was no evidence of acquired surface dyslexia in the sample and thus the present study found no evidence for the double dissociation. However, the possibility of acquired surface dyslexia in Slovakian should not be ruled out. This is because the choice of irregular words was not ideal. Items were not matched for word frequency, length, concreteness or imageability. We know that regularization errors are most common to irregular words in English and French that are low in frequency and imageability. Therefore the present items may not be sensitive to regularistion errors It is necessary to use words that do not follow grapheme to phoneme correspondence rules to identify surface dyslexia in English and French. It will be difficult therefore to detect surface dyslexia in Slovakian. However, other tasks might be used including lexical decision with foreign and loan words. In Italian Toraldo et al. [37] measured positioning of major stress in words of three or more syllables. They assumed that information regarding stress position cannot be accessed through a sub-lexical reading mechanism, as it is unpredictable and not diacritically marked. Similarly Janssen [23] measured stress assignment in patients with surface dyslexia in German. In Slovak there is a stress rule that indicates the stress position in the first syllable irrespective of word length. There are exceptions to this rule and these can be investigated in relation to sublexical processing and hence acquired surface dyslexia in Slovakian. A further point to note, however, that irregular word reading ability is highly correlated with reading experience in English. Therefore, prior exposure to loan and foreign words and irregular stress assignment will need to be controlled in Slovakian speakers.

There were a relatively small number of semantic errors in Slovakian reading and only one participant displayed the characteristics of deep dyslexia. Semantic errors might be rare because of the small sample size and number of stimuli, reducing the possibility that those semantic errors are no more than chance events [16]. A more comprehensive word list is needed to survey the effects of concreteness, imageability and frequency. Two frequency dictionaries are available in Slovakian: one based on estimates from 1969 [30], and the other from 2005 [36]. However a more comprehensive word corpus similar to the large scale databases available for English and French is necessary to detect frequency effects.

The classical account of deep dyslexia assumes two pathways for oral reading [28]. The lexical-semantic pathway maps whole written words to their representations of meaning. These are then mapped to phonological representation for spoken word output. The sublexical pathway parses words into letters or letter groups and maps them to corresponding sounds [21]. Semantic errors are assumed to arise because damage to the sublexical pathway removes the constraint of phonological control over the semantic pathway. We argued above that the sublexical reading mechanism in Slovakian is selectively impaired in patients with phonological dyslexia. One question that follows is why damage to the sublexical reading mechanism did not cause more semantic errors in Slovakian speakers? Semantic reading errors are observed in English even when a patient can implicitly process phonological information via sublexical reading mechanisms [26]. This shows that very limited damage to the reading system is enough to generate semantic errors in an opaque orthography possibly because reading of irregular words and homophones requires frequent and regular use of a lexical-semantic pathway. By contrast, semantic errors in transparent scripts [2] may only arise following severe damage to sublexical mechanisms [29]. This would allow partial phonological information produced from the damaged sublexical pathway to prevent semantic errors in Slovakian even if it is not sufficient to support nonword reading [19]. Note from Table 3 that patients who read nonwords very poorly produced semantic errors. This correlation suggests that damage to sublexical reading mechanisms in Slovakian removes the constraint of phonological control over semantic errors. Another possibility is that reading via the lexical pathway and thus the production of semantic errors in Slovakian is limited to very proficient readers who develop strong links between orthography and meaning prior to brain damage. Although there is no evidence that the participants who produced semantic errors in this study were premorbidly better readers, it will be important for future studies of acquired dyslexia in Slovakian to consider individual differences in premorbid reading style.

In conclusion, the present study found that Slovakian-speaking aphasics show patterns of reading disorder that are similar to phonological dyslexia and deep dyslexia in English and French. However there was no evidence of surface dyslexia. These findings show that reading in Slovakian depends on both a lexical-semantic and a sublexical reading mechanism and that orthographic transparency constrains the relative contribution of lexical and sublexical mechanisms in oral reading [4]. Therefore the strongest prediction of the orthographic depth hypothesis, which assumes a sublexical reading mechanism is sufficient to read in Slovakian, can be rejected. The division of labour for reading in Slovakian is likely to be biased toward the sublexical reading pathway and may rarely engage the lexical-semantic pathway. For this reason semantic reading errors in Slovakian may only follow severe damage to the sublexical reading mechanism and errors resembling surface dyslexia will be qualitatively different to errors in English and French.
